# Dabrafenib protects from cisplatin-induced hearing loss in a clinically relevant mouse model

**DOI:** 10.1172/jci.insight.171140

**Published:** 2023-12-22

**Authors:** Matthew A. Ingersoll, Richard D. Lutze, Chithra K. Pushpan, Regina G. Kelmann, Huizhan Liu, Mark T. May, William J. Hunter, David Z.Z. He, Tal Teitz

**Affiliations:** 1 Department of Pharmacology and Neuroscience,; 2 Department of Biomedical Sciences, and; 3 Department of Pathology, School of Medicine, Creighton University, Omaha, Nebraska, USA.

**Keywords:** Otology, Therapeutics, Cancer, Drug therapy, Protein kinases

## Abstract

The widely used chemotherapy cisplatin causes permanent hearing loss in 40%–60% of patients with cancer. One drug, sodium thiosulfate, is approved by the FDA for use in pediatric patients with localized solid tumors for preventing cisplatin-induced hearing loss, but more drugs are desperately needed. Here, we tested dabrafenib, an FDA-approved BRAF kinase inhibitor and anticancer drug, in a clinically relevant multidose cisplatin mouse model. The protective effects of dabrafenib, given orally twice daily with cisplatin, were determined by functional hearing tests and cochlear outer hair cell counts. Toxicity of the drug cotreatment was evaluated, and levels of phosphorylated ERK were measured. A dabrafenib dose of 3 mg/kg BW, twice daily, in mice, was determined to be the minimum effective dose, and it is equivalent to one-tenth of the daily FDA-approved dose for human cancer treatment. The levels of hearing protection acquired, 20–25 dB at the 3 frequencies tested, in both female and male mice, persisted for 4 months after completion of treatments. Moreover, dabrafenib exhibited a good in vivo therapeutic index (> 25), protected hearing in 2 mouse strains, and diminished cisplatin-induced weight loss. This study demonstrates that dabrafenib is a promising candidate drug for protection from cisplatin-induced hearing loss.

## Graphical abstract

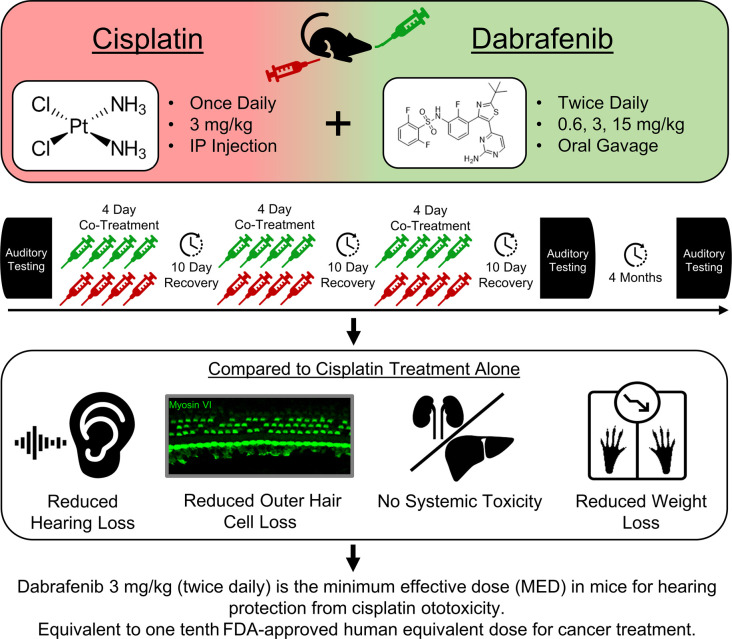

## Introduction

Cisplatin is a highly effective and commonly used chemotherapy agent for the treatment of a variety of cancers, but 40%–60% of patients treated with cisplatin have irreversible hearing loss (1, 2). Cisplatin negatively affects high-frequency hearing more than lower frequencies primarily due to death of outer hair cells (OHCs) in the cochlear basal turn (3, 4). Hair cells are the most common cochlear cell type to be affected by cisplatin, but cells of the stria vascularis, spiral ganglion neurons, and supporting cells have also been reported to suffer deleterious effects (5, 6). Cisplatin-induced hearing loss negatively impacts an individual’s quality of life, leading to depression and social isolation (7), and impedes the development of language skills in young children treated with cisplatin (1–3). There is a dire clinical need to develop drugs that can protect from this highly common side effect of cisplatin treatment. Currently, there is only 1 FDA-approved drug for the treatment of cisplatin ototoxicity, which has limited application. Sodium thiosulfate (STS), brand name Pedmark, was recently approved by the FDA to reduce the risk of cisplatin-induced ototoxicity in pediatric patients 1 month or older with localized, nonmetastatic solid tumors and represents a significant advancement in the field of hearing loss prevention (8–11). STS is administered to patients 6 hours after cisplatin treatment due to concerns over its interference with cisplatin’s tumor-killing efficacy, even though no conclusive data demonstrate direct interference. No difference in hearing outcomes is observed with the delay in treatment (10–17). Recently, the antioxidant N-acetylcysteine (NAC) was shown to be otoprotective in a phase I clinical trial in children and adolescents diagnosed with localized, nonmetastatic, cisplatin-treated tumors (18). No severe adverse events occurred following NAC treatment, which makes it a promising compound for the treatment of cisplatin-induced hearing loss. While the approval of STS and the clinical testing of NAC are beneficial for the treatment of cisplatin-induced hearing loss for localized solid tumors in pediatric patients, there remains a clear therapeutic need to develop additional drugs that can protect from cisplatin ototoxicity for adults and children who do not meet the requirements for Pedmark treatment, such as patients with metastatic disease.

Our laboratory has recently demonstrated that dabrafenib (Tafinlar), an FDA-approved BRAF inhibitor, was a top hit in a high-throughput cell-based screen of an inner ear cell line for protection from cisplatin-induced cell death (6). In addition, dabrafenib protected OHCs in neonatal mouse cochlear explants from cisplatin-induced death with an IC_50_ of 30 nM and a therapeutic index larger than 2,000. Importantly, dabrafenib mitigated cisplatin-induced hearing loss and OHCs’ death in adult mice at clinically relevant doses (100 mg/kg body weight [BW], once daily) (6, 19). These experiments were performed with cisplatin administered once at a single high dose of 30 mg/kg BW in FVB/NJ mice. This high dose of cisplatin was necessary to inflict hearing loss in FVB/NJ mice with threshold shifts of 20–25 dB sound pressure level (SPL) at 8, 16, and 32 kHz (6, 20).

BRAF is a member of the Raf family of protein kinases, which is upstream of MEK and ERK in the canonical signal transduction pathway called the mitogen-activated protein kinase (MAPK) pathway (21). MAPK proteins are activated when they are phosphorylated, and dabrafenib prevents BRAF from phosphorylating downstream MEK. This significantly lowers the activity of the MAPK pathway. This pathway has been extensively studied in the cancer field, and approximately one-third of all cancers have dysregulated MAPK activity (22). MAPK activation is known to be involved in cell proliferation and cell survival, but it has a different role in postmitotic cells, including cells in the inner ear (23–34). Our laboratory showed that dabrafenib’s mechanism of protection was through inhibition of the MAPK pathway, which is upregulated in the inner ear following cisplatin administration, but cotreatment with dabrafenib decreased MAPK activity and protected hair cells from cisplatin-induced death (6). Importantly, 6 other drugs in the BRAF/MEK/ERK pathway protect against cisplatin-induced hair cell death in mouse cochlear explants (6). MAPK activation after cisplatin administration was most notably observed in the inner ear supporting cells but was also seen in the spiral ganglion neurons and nerve fibers that innervate the hair cells (6).

Dabrafenib was first approved by the US FDA for metastatic melanoma in 2013 and thyroid cancer in 2018 as well as the EU for non–small cell lung carcinoma in 2017 (35, 36). Patients who receive dabrafenib treatment have the activated BRAF V600E or V600K mutations that are present in half of all patients with metastatic melanoma (36). In June 2022, the FDA granted accelerated approval to dabrafenib in combination with trametinib for the treatment of nearly all adult and pediatric patients above 6 years of age with unresectable or metastatic solid tumors with the BRAF V600E mutation who have progressed following prior treatment and have no satisfactory alternative therapeutic options (37).

There are many advantages for repurposing dabrafenib, a widely used anticancer drug, as a therapeutic compound to protect patients from cisplatin-induced hearing loss: (i) Dabrafenib is a well-tolerated drug with a good therapeutic window that is given to patients daily for up to a year. In human patients, relatively minor side effects are observed, such as fever, joint pain, skin rash, and papilloma (38). In our hearing studies, mice did not exhibit any deleterious toxicity or ototoxic side effects from dabrafenib treatment (6). (ii) Dabrafenib is given orally, which is an easy administration route for patients inside and outside a clinical setting (39). (iii) Dabrafenib does not interfere with cisplatin’s tumor-killing ability in 6 cell lines from 2 types of tumors for which cisplatin is the standard of care: neuroblastoma and lung cancer (6). (iv) Dabrafenib is already FDA approved, and FDA-approved drugs have much shorter developmental times as the absorption, distribution, metabolism, excretion, and toxicity properties of the drugs in humans are already known (40). Thus, the cost of developing the drugs is up to 40% less to bring to market compared with non–FDA-approved drugs (41). Recently, FDA-approved drugs, such as metformin used to treat diabetes and atorvastatin used to lower cholesterol, have been tested for hearing protection and have entered clinical trials (42–44). (v) Dabrafenib crosses the blood-brain barrier, which is similar to the blood-labyrinth barrier, and has shown protection in our mouse models from cisplatin-induced hearing loss (6, 45).

Recently, Fernandez et al. and Roy et al. developed a clinically relevant mouse model to study cisplatin-induced hearing loss (46, 47). In this model, mice are treated with a low dose of cisplatin, 3 mg/kg BW, for 4 days, which is then followed with a 10-day recovery period. This cycle is then repeated for a total of 3 times. This new mouse treatment protocol mimics the treatment paradigm used for humans. Previously, our laboratory has utilized a single, high-dose cisplatin (30 mg/kg) treatment protocol to establish the protective effect of dabrafenib. Dabrafenib significantly protected mice from cisplatin-induced hearing loss when given at a dose of 100 mg/kg daily for 3 days via oral gavage (6). However, human patients are typically given multiple low doses of cisplatin over a week and in monthly cycles and not in a high, single dose (48). Additionally, cisplatin-treated CBA/CaJ mice in the multicycle protocol have greater hearing loss compared with FVB/NJ mice treated with a single high dose. Moreover, there is minimal mouse death in this multicycle protocol from cisplatin treatment while significant mouse death occurs in the single-dose protocol (46). The similarity of this mouse model to the cisplatin protocol that patients receive allows for more translational conclusions to be drawn.

In this study, we tested in the single, high-dose cisplatin mouse protocol a 1:4 lower dose of dabrafenib (12 mg/kg, twice daily) than our previous published studies (6) and 3 de-escalating doses of dabrafenib in the multicycle cisplatin mouse regimen. Three different functional hearing tests were performed to determine dabrafenib’s ability to protect from cisplatin-induced hearing loss: 1) the auditory brainstem response (ABR) is utilized to measure overall hearing function in the mice; 2) distortion product otoacoustic emission is performed to determine whether dabrafenib protects from OHC dysfunction, which occurs with cisplatin treatment; and 3) the endocochlear potential (EP) is used to measure whether dabrafenib protects the stria vascularis from cisplatin-induced damage. Previous studies have implicated that damage to the stria vascularis could be one of the main reasons hearing loss and hair cell death occur from cisplatin treatment (5, 49–51). Additionally, OHC counts are performed to measure dabrafenib’s ability to protect from cisplatin-induced hair cell death. We also tested whether phosphorylation of ERK, downstream of BRAF, is upregulated in the cochlear cells with cisplatin treatment in the multidose cisplatin regimen and downregulated with dabrafenib cotreatment as we evidenced in the single, high-dose cisplatin protocol (6). Finally, total mouse weight measurements and histological studies of the kidney and liver, 2 main organs in which cisplatin accumulates and causes damage, are examined to ensure dabrafenib treatment in combination with cisplatin does not cause additional toxicity. Overall, the combined results of this study show that oral treatment with dabrafenib is a promising and effective therapeutic strategy to protect from cisplatin-induced hearing loss.

## Results

### Dabrafenib protects against cisplatin-induced hearing loss in a single, high-dose cisplatin mouse model.

**Figure 1:**
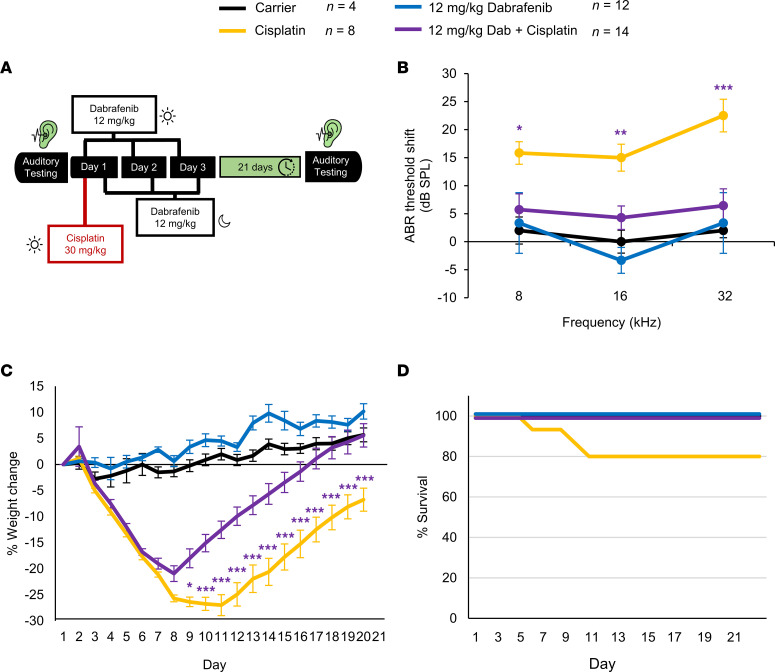
Dabrafenib protects against cisplatin-induced hearing loss following a single high dose of cisplatin. (**A**) Schedule of administration of dabrafenib and cisplatin in FVB mice: 30 mg/kg cisplatin was administered once on day 1 while 12 mg/kg dabrafenib was administered for 3 days, twice a day. Auditory testing was performed before treatment began and 21 days after cisplatin administration. (**B**) ABR threshold shifts following protocol in **A**. (**C**) Weight change over 21 days following protocol in **A**. (**D**) Kaplan-Meier survival curves of mouse cohorts following protocol in **A**. Carrier alone (black), cisplatin alone (yellow), dabrafenib alone (blue), and dabrafenib plus cisplatin (purple). Data shown as means ± SEM; *P < 0.05, **P < 0.01, ***P < 0.001 compared with cisplatin alone by 2-way ANOVA with Bonferroni’s post hoc test.

Previous studies from our lab demonstrated that the BRAF inhibitor dabrafenib, administered by oral gavage at 100 mg/kg BW, once daily for 3 consecutive days, protected FVB/NJ adult mice against a single, high-dose cisplatin (30 mg/kg) intraperitoneal injection that causes permanent hearing loss in this mouse strain (6, 20). In the current study, we first tested a lower dose of 12 mg/kg dabrafenib using the single-dose cisplatin protocol to compare it with the previously used 100 mg/kg dabrafenib dose (6). Dabrafenib was administered twice daily by oral gavage, for 3 consecutive days, with the first dose given 45 minutes before cisplatin injection (Figure 1A). Dabrafenib provided significant protection from cisplatin-induced hearing loss by ABR functional hearing measurements at 8, 16, and 32 kHz frequencies, with the greatest protection observed at 32 kHz (Figure 1B). The average protection achieved was 10 dB SPL at 8 kHz, 10 dB at 16 kHz, and 16 dB at 32 kHz. Twice-daily 12 mg/kg dabrafenib (40% of the human equivalent dose) (19) provided equivalent hearing protection to the previously tested once-daily 100 mg/kg dose (Figure 1B). Interestingly, mice administered both dabrafenib and cisplatin experienced a significant reduction in weight loss, beginning on day 9 and persisting through day 21, compared with the cisplatin alone–treated cohort, while those treated with dabrafenib alone exhibited no change in weight compared with carrier alone (Figure 1C). Additionally, no mouse death occurred in cohorts treated with dabrafenib and cisplatin, while 20% of mice treated with cisplatin alone died (Figure 1D).

### Dabrafenib protects against cisplatin-induced hearing loss using a multicycle, low-dose cisplatin treatment regimen.

**Figure 2:**
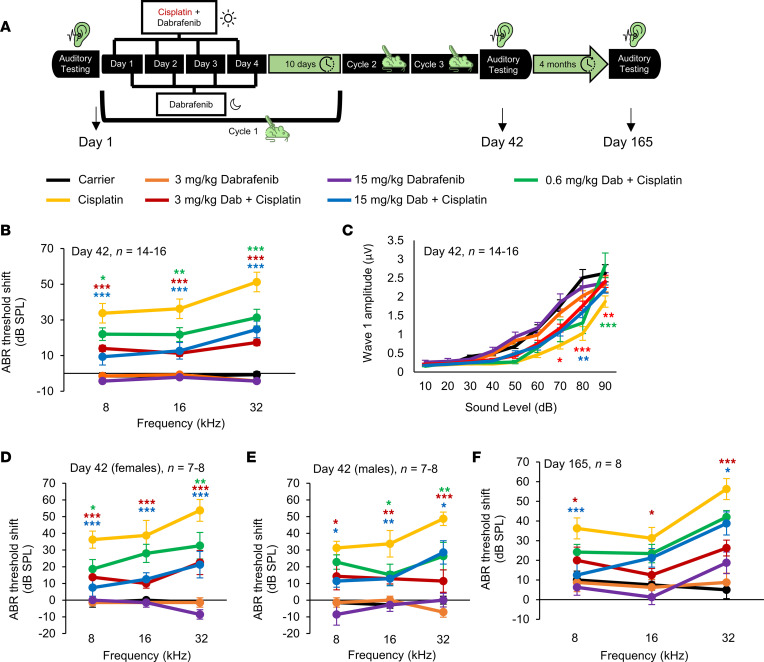
Dabrafenib-treated mice have significantly lower ABR threshold shifts compared with cisplatin alone–treated mice. (**A**) Schedule of administration of dabrafenib and cisplatin in a translational, multicycle treatment protocol using CBA/CaJ mice. Each cycle consisted of 4 days of treatment with 3 mg/kg cisplatin in the morning and 15, 3, or 0.6 mg/kg dabrafenib in the morning and evening. A 10-day recovery period followed the 4 days of treatment. This cycle was repeated for a total of 3 times. Auditory testing occurred before treatment began, immediately after cycle 3 (day 42), and 4 months after cycle 3 (day 165). (**B**) ABR threshold shifts recorded immediately after the completion of cycle 3 (day 42) in protocol shown in **A**. (**C**) Amplitudes of ABR wave 1 at 16 kHz from **B**. (**D**) ABR threshold shifts of female and (**E**) male mice recorded immediately after the completion of cycle 3. (**F**) ABR threshold shifts recorded 4 months after the completion of cycle 3 (day 165). Carrier (black), cisplatin alone (yellow), 15 mg/kg dabrafenib alone (purple), 3 mg/kg dabrafenib alone (orange), 15 mg/kg dabrafenib plus cisplatin (blue), 3 mg/kg dabrafenib plus cisplatin (red), and 0.6 mg/kg dabrafenib plus cisplatin (green). Data shown as means ± SEM; *P < 0.05, **P < 0.01, ***P < 0.001 compared with cisplatin alone by 2-way ANOVA with Bonferroni’s post hoc test.

Human patients treated with cisplatin are not administered a single high dose (48). We therefore sought to test the efficacy of dabrafenib to protect from cisplatin ototoxicity in a clinically relevant mouse model following a protocol initially developed by Roy et al. in 2013 and optimized by Fernandez at al. in 2019 (Figure 2A) (46, 47). The doses of dabrafenib tested in this study are 15, 3, and 0.6 mg/kg. We chose 15 mg/kg as it is close to the lowest effective dose tested of dabrafenib in the high, single-dose cisplatin protocol (12 mg/kg, Figure 1, B and C) and 2 additional 1:5 deescalating doses (3 and 0.6 mg/kg) to determine the drug’s minimum effective dose. Dabrafenib at doses of 15 or 3 mg/kg BW provided significant protection from cisplatin-induced hearing loss in this clinically relevant mouse model. As shown in Figure 2B and Supplemental Figure 2, mice cotreated with 15, 3, and 0.6 mg/kg dabrafenib and cisplatin had significantly lower ABR threshold shifts compared with cisplatin alone–treated mice, with an ABR average threshold shift reduction at 32 kHz of 27, 34, and 20 dB, respectively. Mice treated with 3 mg/kg dabrafenib had significantly higher ABR wave 1 amplitudes at 16 kHz compared with cisplatin alone at 90, 80, and 70 dB SPL, while 15 mg/kg had significantly higher wave 1 amplitude at 80 dB SPL and 0.6 mg/kg at 90 dB SPL (Figure 2C). Additionally, mice cotreated with 15 or 3 mg/kg dabrafenib and cisplatin had lower ABR threshold shifts for both males and females at the 8, 16, and 32 kHz frequency regions. Male mice treated with 0.6 mg/kg had significantly lower threshold shifts at 8 and 32 kHz and females at 16 and 32 kHz (Figure 2, D and E). Furthermore, the hearing protection of mice given 15 or 3 mg/kg dabrafenib did not diminish 4 months after the completion of the 42 days of treatment, with significant protection maintained at all frequencies tested. The 0.6 mg/kg dabrafenib treated mice lost their protection at this time point (Figure 2F). No statistically significant difference in ABR threshold shifts was observed between the 15 mg/kg dabrafenib cotreated group and the 3 mg/kg dabrafenib cotreated group (Figure 2F).

**Figure 3:**
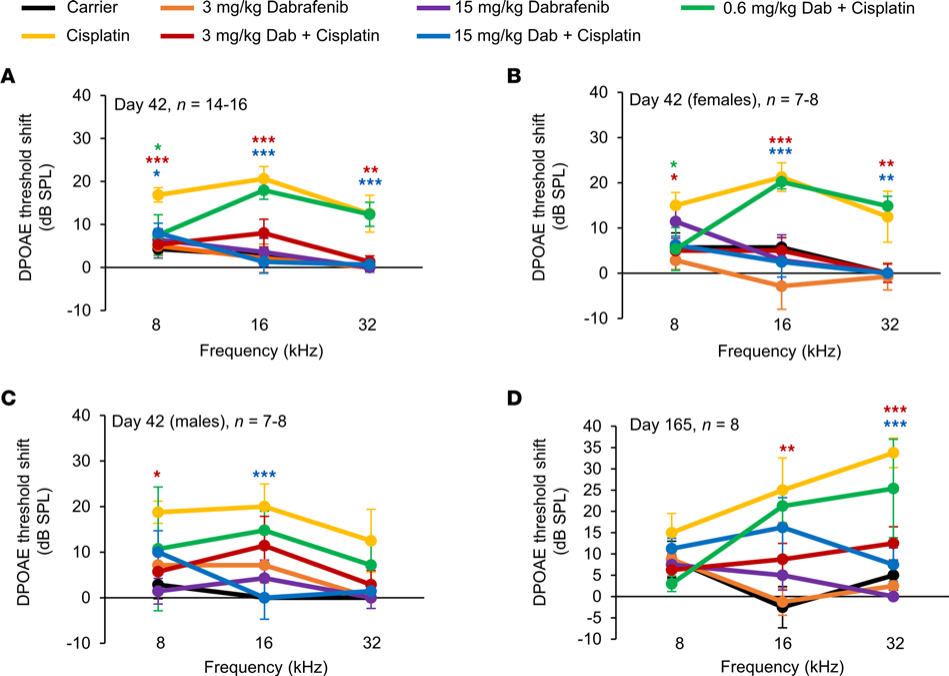
Dabrafenib-treated mice have significantly lower DPOAE threshold shifts compared with cisplatin alone–treated mice. (**A**) DPOAE threshold shifts recorded immediately after the completion of cycle 3 (day 42) in protocol shown in Figure 2A. (**B**) DPOAE threshold shifts of female and (**C**) male mice recorded immediately after the completion of cycle 3. (**D**) DPOAE threshold shifts recorded 4 months after the completion of cycle 3 (day 165). Carrier (black), cisplatin alone (yellow), 15 mg/kg dabrafenib alone (purple), 3 mg/kg dabrafenib alone (orange), 15 mg/kg dabrafenib plus cisplatin (blue), 3 mg/kg dabrafenib plus cisplatin (red), and 0.6 mg/kg dabrafenib plus cisplatin (green). Data shown as means ± SEM; *P < 0.05, **P < 0.01, ***P < 0.001 compared with cisplatin alone by 2-way ANOVA with Bonferroni’s post hoc test.

Distortion product otoacoustic emission (DPOAE) threshold shifts were also calculated immediately after and 4 months following the completion of cycle 3. As shown in Figure 3A, mice cotreated with 15 or 3 mg/kg dabrafenib and cisplatin had significantly lower DPOAE threshold shifts compared with the cisplatin alone–treated mice, with a reduction in average DPOAE threshold shifts at 16 kHz of 19 and 13 dB SPL, respectively. Cotreatment of cisplatin and dabrafenib at 0.6 mg/kg had significantly lower DPOAE threshold shift at 8 kHz only immediately after the completion of cycle 3 (Figure 3A). Males and females were analyzed separately, and dabrafenib-cotreated mice with cisplatin had significantly lower DPOAE threshold shifts in both sexes (Figure 3, B and C). The 3 mg/kg dabrafenib cotreatment with cisplatin had significantly lower DPOAE threshold shifts at all 3 tested frequencies in females, while males had significantly lower threshold shifts at 8 kHz. The 15 mg/kg dabrafenib cotreatment with cisplatin had significantly lower DPOAE threshold shifts at the 16 and 32 kHz frequencies in females and 16 kHz in males. The 0.6 mg/kg dabrafenib cotreatment with cisplatin had significantly lower DPOAE threshold shifts at 8 kHz in females only (Figure 3, B and C). DPOAE threshold shifts measured at 4 months after the completion of cycle 3 showed mice cotreated with 3 mg/kg dabrafenib and cisplatin had significantly lower threshold shifts at 16 and 32 kHz, while 15 mg/kg dabrafenib- and cisplatin-treated mice had significance at 32 kHz (Figure 3D).

**Figure 4:**
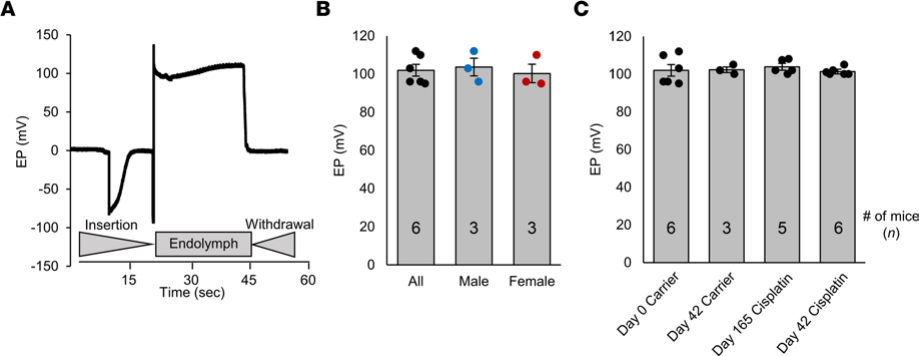
EP remains unchanged after cisplatin treatment. (**A**) Representative EP measured from a CBA/CaJ mouse. The times of insertion into the endolymph and withdrawal are shown below the trace. (**B**) Average EP measurements from mice before the treatment protocol in Figure 2A began. Additionally, males and females are graphed individually. (**C**) Average EP measurements of mice treated with carrier or cisplatin at different time points throughout protocol. Groups from left to right are as follows: untreated mice before protocol began, carrier-treated mice measured immediately after cycle 3 (day 42), cisplatin-treated mice measured immediately after cycle 3, and cisplatin-treated mice measured 4 months after cycle 3 (day 165). Data shown as means ± SEM; all groups compared with one another by 2-way ANOVA with Bonferroni’s post hoc test.

The last functional test was EP to determine whether cisplatin caused functional damage to the stria vascularis after the multicycle cisplatin protocol. Figure 4A shows an example EP recording depicting the microelectrode insertion and withdrawal from the scala media through the basilar membrane (organ of Corti) (52, 53). Before any treatment began, EP from 6 mice were recorded with an average potential of 103 mV with no difference between males and females (Figure 4B). EP was recorded again in carrier and cisplatin alone–treated mice immediately and 4 months after the completion of cycle 3. There was no change in EP for mice treated with cisplatin at all time points tested (Figure 4C).

### Dabrafenib protects against cisplatin-induced OHC loss.

**Figure 5:**
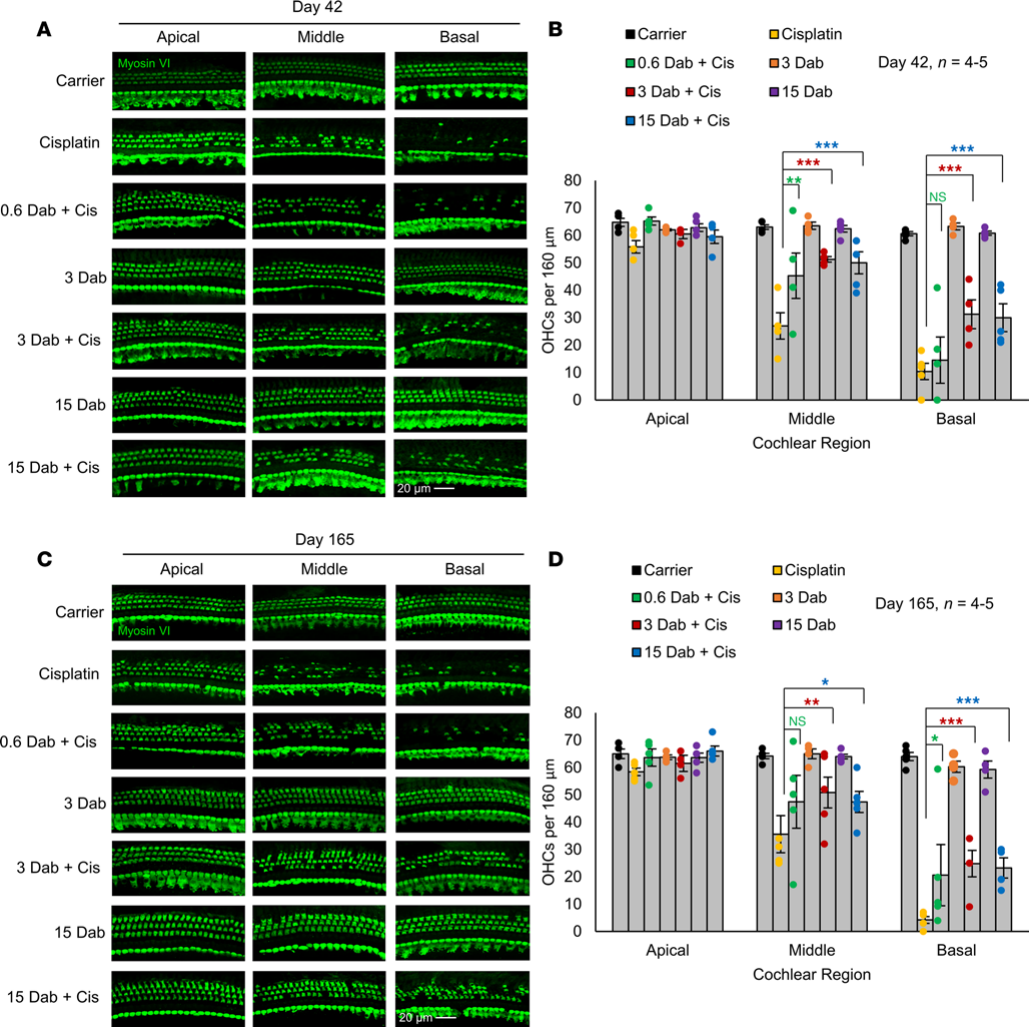
Dabrafenib protects from cisplatin-induced OHC death. (**A**) Representative myosin VI–stained confocal images of the 8, 16, and 32 kHz regions of the cochlea collected immediately after the completion of cycle 3 (day 42) of protocol shown in Figure 2A. (**B**) Number of OHCs per 160 μm at the 8, 16, and 32 kHz regions of cochlea collected immediately after the completion of cycle 3. (**C**) Representative myosin VI–stained confocal images of the 8, 16, and 32 kHz regions of the cochlea collected 4 months after the completion of cycle 3 (day 165). (**D**) Number of OHCs per 160 μm at the 8, 16, and 32 kHz regions of cochlea collected 4 months after the completion of cycle 3. Carrier (black), cisplatin alone (yellow), 15 mg/kg dabrafenib alone (purple), 3 mg/kg dabrafenib alone (orange), 15 mg/kg dabrafenib plus cisplatin (blue), 3 mg/kg dabrafenib plus cisplatin (red), and 0.6 mg/kg dabrafenib plus cisplatin (green). Data shown as means ± SEM; *P < 0.05, **P < 0.01, ***P < 0.001 compared with cisplatin alone by 2-way ANOVA with Bonferroni’s post hoc test. *n* = 4–5.

After all functional tests were performed, cochleae were dissected for analysis of OHCs. At day 42, mice cotreated with 15 and 3 mg/kg dabrafenib and cisplatin had significantly more OHCs at the basal region compared with cisplatin alone–treated mice, while 15, 3, and 0.6 mg/kg dabrafenib also had significantly more OHCs at the middle region (Figure 5, A and B). Cisplatin alone–treated mice had a mean ± SEM of 36 ± 7 and 4 ± 1 OHCs following cisplatin treatment at the 16 and 32 kHz regions per 160 μm, respectively. At the 16 and 32 kHz regions, mice treated with 15 mg/kg dabrafenib had 47 ± 4 and 23 ± 4 OHCs following treatment, while mice treated with 3 mg/kg dabrafenib had 51 ± 6 and 25 ± 5 OHCs per 160 μm, respectively. The 0.6 mg/kg dabrafenib-treated mice had slightly fewer OHCs compared with mice at the higher dabrafenib doses, 47 ± 10 at 16 kHz and 21 ± 11 at 32 kHz. At day 165, 15 and 3 mg/kg treated mice had significantly more OHCs at the basal and middle region of the cochlea compared with the cisplatin alone–treated mice. The dose of 0.6 mg/kg dabrafenib conferred protection from OHC loss at the middle region but not at the basal region (Figure 5, C and D). At the 16 and 32 kHz regions, cisplatin alone–treated mice had 27 ± 5 and 10 ± 3 OHCs per 160 μm, while mice treated with 15 mg/kg dabrafenib had 50 ± 4 and 30 ± 5 OHCs, respectively. The mice treated with 3 mg/kg dabrafenib had 51 ± 1 OHCs at the 16 kHz region and 31 ± 5 at the 32 kHz region, while 0.6 mg/kg dabrafenib-treated mice had 45 ± 8 OHCs at 16 kHz and 15 ± 8 at 32 kHz.

### Dabrafenib mitigates cisplatin-induced phosphorylation of ERK.

**Figure 6:**
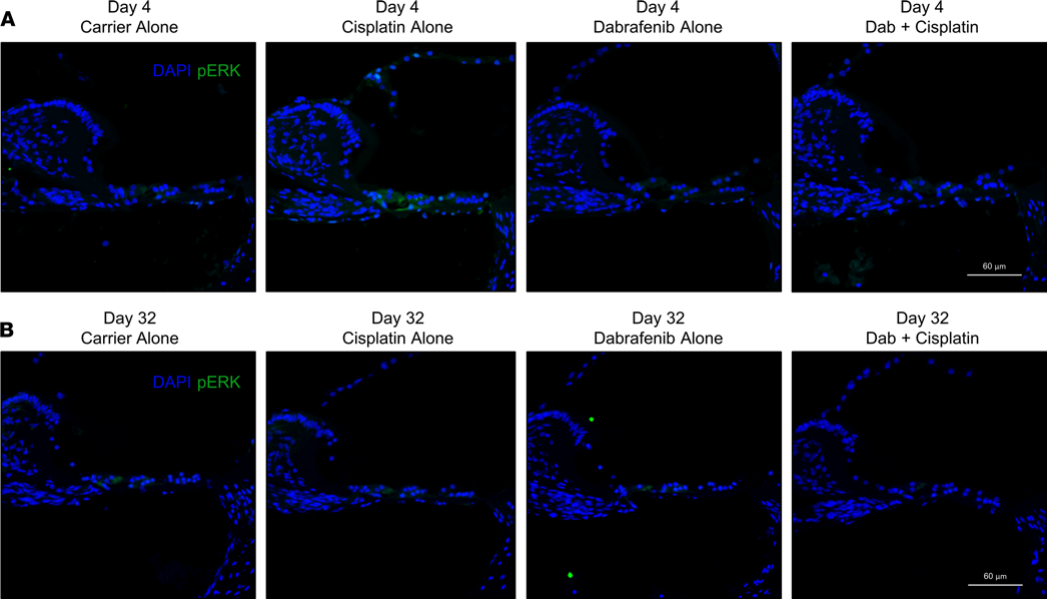
Dabrafenib attenuates ERK phosphorylation in the cochlear organ of Corti during the multicycle cisplatin treatment protocol. (**A**) Representative images of cochlear cryosections stained with DAPI (blue) and phosphorylated ERK (green) on day 4 of the protocol in Figure 2A. Mice were sacrificed 45 minutes following the last cisplatin injection of cycle 1. Total *n* = 3 mice from each experimental group were tested. (**B**) Representative images of cochlear cryosections on day 32. Mice were sacrificed 45 minutes following the last cisplatin injection of cycle 3. Experimental groups from left to right are as follows: carrier alone, cisplatin alone, 3.0 mg/kg dabrafenib alone, and 3.0 mg/kg dabrafenib + cisplatin. Total *n* = 3 mice from each experimental group were tested. Scale bars: 60 μm.

Cochleae were collected from mice at the end of treatment cycles 1 and 3, day 4 and 32, respectively, to examine cisplatin and dabrafenib’s effect on phosphorylation of the downstream target ERK. On day 4, cisplatin-treated mice had increased ERK phosphorylation in the organ of Corti region of the middle turn compared with other cohorts. Cotreatment of 3 mg/kg dabrafenib with cisplatin mitigated phosphorylation of ERK in the organ of Corti; similarly, elevated phosphorylation of ERK was not observed in carrier- and 3 mg/kg dabrafenib–treated mice (Figure 6A). Changes in ERK phosphorylation were not observed in other regions of the cochleae, including the stria vascularis, spiral limbus, spiral ligament, and spiral ganglion neurons, on day 4. Increased ERK phosphorylation was not observed in any cohort on day 32, including cisplatin-treated mice (Figure 6B). Together, the data demonstrate cisplatin induces phosphorylation of ERK in the organ of Corti early in cycle 1 and that dabrafenib cotreatment mitigates this change in MAPK signaling.

### Dabrafenib does not increase systemic toxicity when combined with cisplatin.

**Figure 7:**
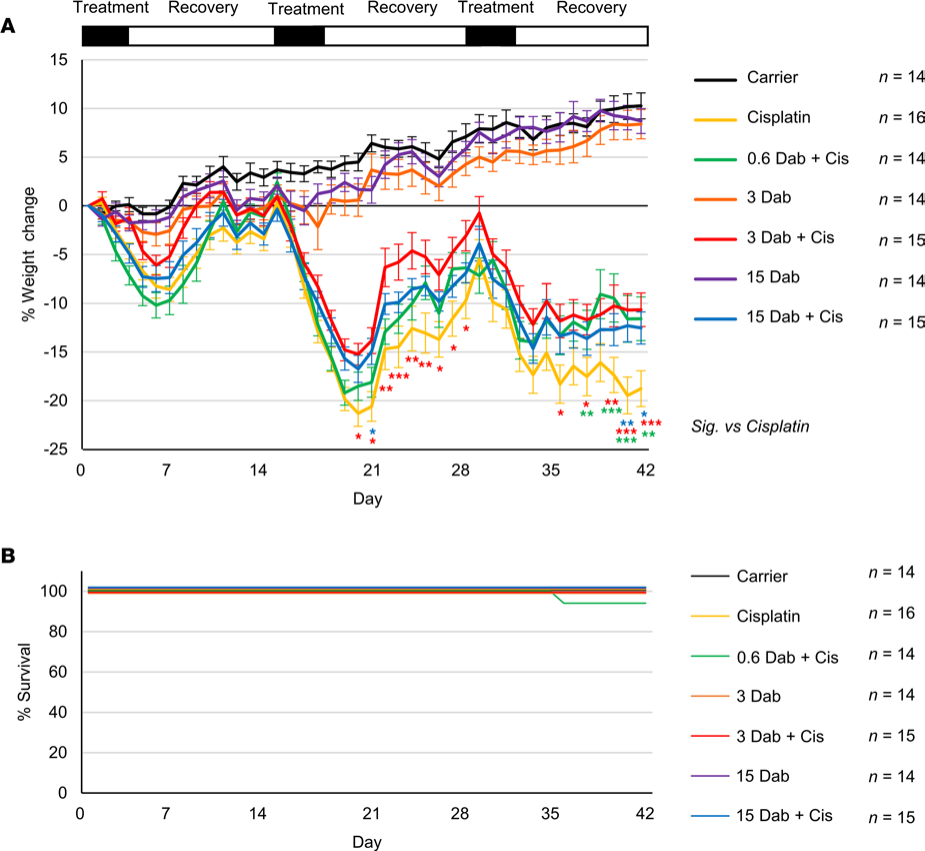
Dabrafenib-treated mice have less weight loss during the multicycle cisplatin protocol. (**A**) Weight loss over the 42-day treatment protocol shown in Figure 2A. Carrier (black), cisplatin alone (yellow), 15 mg/kg dabrafenib alone (purple), 3 mg/kg dabrafenib alone (orange), 15 mg/kg dabrafenib plus cisplatin (blue), 3 mg/kg dabrafenib plus cisplatin (red), and 0.6 mg/kg dabrafenib plus cisplatin (green). (**B**) Kaplan-Meier survival curves of mouse cohorts going to day 42 following protocol in Figure 2A. Data shown as means ± SEM; *P < 0.05, **P < 0.01, ***P < 0.001 compared with cisplatin alone by 2-way ANOVA with Bonferroni’s post hoc test.

Throughout the multicycle treatment protocol, mice are weighed daily to analyze weight loss for each cohort. Cisplatin-treated mice lost up to 21% body weight throughout the treatment regimen. Carrier-treated and dabrafenib (3 and 15 mg/kg) alone–treated mice did not exhibit weight loss, but rather steadily gained weight. All 3 doses of dabrafenib (15, 3, and 0.6 mg/kg) showed significantly less weight loss on multiple days in mice cotreated with cisplatin compared to cisplatin alone (Figure 7A). Dabrafenib at 3 mg/kg demonstrated the best protection from weight loss, with cotreated mice losing only 15% of original body weight throughout both cycles 2 and 3 (Figure 7A). Mice were again weighed on day 165, and all cohorts exhibited similar weights, with no significant difference between groups (Supplemental Figure 1A; supplemental material available online with this article; https://doi.org/10.1172/jci.insight.171140DS1). There was no significant mouse death in any treatment group throughout the protocol (Figure 7B and Supplemental Figure 1B).

**Figure 8:**
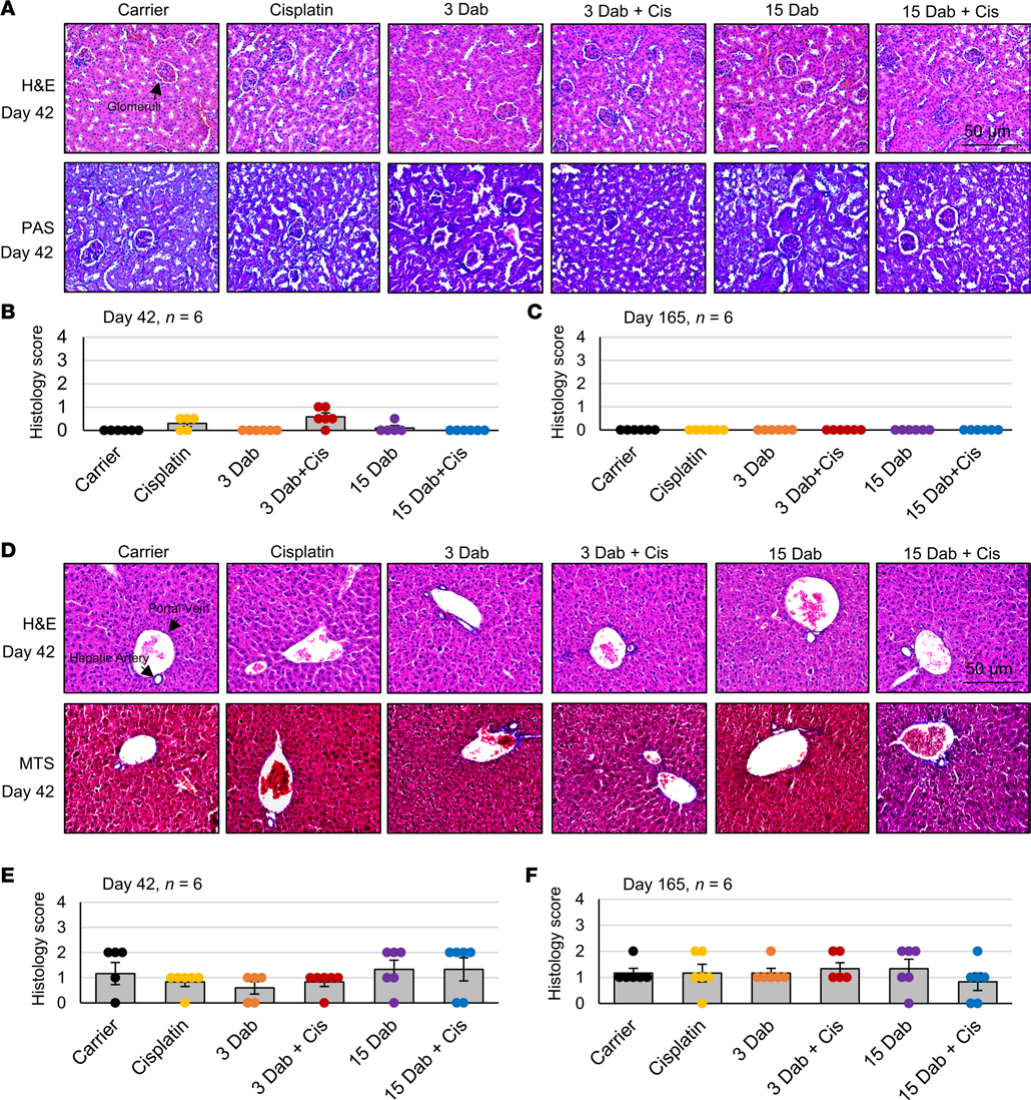
Dabrafenib and cisplatin do not cause significant damage to the kidneys or liver. (**A**) Representative H&E and PAS images of the kidney at 20× original magnification. Treatment groups from left to right are as follows: carrier alone, cisplatin alone, 3 mg/kg dabrafenib alone, 3 mg/kg dabrafenib plus cisplatin, 15 mg/kg dabrafenib alone, and 15 mg/kg dabrafenib plus cisplatin. (**B**) Kidneys collected immediately after cycle 3 and (**C**) 4 months after cycle 3 were stained with H&E and PAS and scored in a blinded manner by an experienced pathologist. Score of 0 indicates no visible damage while a score of 4 indicates very severe damage. (**D**) Representative H&E- and Masson’s trichrome– stained images of the liver at 20× original magnification. (**E**) Histology scores of liver samples collected immediately after cycle 3 and (**F**) 4 months after cycle 3 (165 days) blindly scored by experienced pathologist. 0 = normal, 1 = mild damage, 2 = moderate damage, 3 = severe damage, and 4 = very severe (fulminant) damage. Data shown as means ± SEM; all groups compared with one another by 2-way ANOVA with Bonferroni’s post hoc test.

Additionally, mouse livers and kidneys were collected to analyze the toxic effect of cisplatin and dabrafenib on these organs. Kidneys were stained with hematoxylin and eosin (H&E) and Periodic acid–Schiff (PAS) with Figure 8A showing representative images for each group immediately after cycle 3 (54, 55). Samples were then analyzed by a trained and experienced pathologist without knowing the experimental conditions to determine the amount of damage in each group. There was no significant kidney damage in any cohort at both days 42 and 165 (Figure 8, B and C, and Supplemental Figure 3). Livers were stained with H&E and Masson’s trichrome stain, with Figure 8D showing representative images at day 42 (56, 57). There was no significant difference in the amount of liver damage between all experimental groups as indicated by the histology score at both days 42 and day 165 (Figure 8, E and F, and Supplemental Figure 4).

## Discussion

Due to the promising clinical potential of dabrafenib in our high, single-dose cisplatin regimen, we sought to test the drug in a multidose cisplatin model, which is more relevant for human treatment (46). Human patients with cancer typically receive a week of daily cisplatin infusions in cycles spaced a few weeks apart (48). In this work, we took advantage of the model developed by Roy et al. and optimized by Fernandez et al. to test dabrafenib’s protection against cisplatin-induced hearing loss (46, 47). Employing a clinically relevant cisplatin protocol and three 1:5 dilutions of the drug dabrafenib (15, 3, 0.6 mg/kg), we conclude that dabrafenib has an average protection of 19 dB at 8 kHz, 25 dB at 16 kHz, and 34 dB at 32 kHz, after cisplatin treatment with a low dose, 3 mg/kg twice daily (Figure 2). Importantly, the dose of 3 mg/kg BW dabrafenib, twice daily, was found to be as effective as the 15 mg/kg BW dose and is approximately one-tenth of the equivalent dabrafenib dose given to human patients with cancer (19, 58). At 15 and 3 mg/kg, dabrafenib exhibited the same hearing protection with no statistically significant difference between the groups. Thus, 3 mg/kg was determined to be the minimal effective dose in this model. The lowest dose tested of 0.6 mg/kg dabrafenib, which is equivalent to one-fiftieth of the human equivalent dose, still demonstrated protection of 12 dB at 8 kHz, 15 dB at 16 kHz, and 20 dB at 32 kHz, yet it is not as effective as 3 or 15 mg/kg dabrafenib (19). The multidose protocol demonstrated a therapeutic window of at least 25 for dabrafenib in vivo. Protection was observed with a dose as high as 15 mg/kg and as low as 0.6 mg/kg. Higher doses of dabrafenib were not tested; however, previous data obtained from the single, high-dose cisplatin protocol demonstrated 100 mg/kg dabrafenib daily was well tolerated (6). A wide therapeutic index is important for the clinical application of dabrafenib to give clinicians flexibility with dosage without toxicity to the patient.

Our previous results with the single, high-dose cisplatin injection in mice showed that phosphorylation of the downstream ERK1/2 kinase is upregulated after cisplatin or noise damage in the inner ear supporting cells, and it is downregulated upon dabrafenib treatment (6). We observe in this study a similar pattern of upregulation in ERK1/2 phosphorylation after the first cycle of cisplatin in the multidose cisplatin protocol on day 4 (Figure 6A), but interestingly, no upregulation in phosphorylated ERK1/2 was detected after cycle 3 of cisplatin on day 32 (Figure 6B). It may be that the MAPK cascade stress pathway is an early molecular pathway activated by cisplatin damage, and it can be suppressed after continuous damage by feedback loop activation of other kinases in the pathway (59–61).

Protection from weight loss in the cisplatin and dabrafenib–cotreated groups, employing either the single-dose protocol or the multidose regimen, is an unexpected and exciting phenomenon in our studies. Dabrafenib significantly reduced the weight loss typically seen in mice during cisplatin treatment and thus helped maintain the general well-being of the animals (Figure 7A). At this stage, we do not know the molecular mechanism for the reduction in weight loss or whether it is involved in modulating the brain appetite pathways (62, 63). It would be exciting to investigate this advantage further. Preliminary data from our laboratory indicate that treatment with dabrafenib can protect the kidneys from cisplatin-induced acute kidney injury in the single-dose cisplatin protocol. This protection can contribute to the healthier state of the animals with dabrafenib cotreatment throughout the multidose cisplatin protocol as well. The weights of the different experimental animal groups were not different at the endpoint of our experiments at day 165, which agrees with our histological analysis that no significant damage is seen in the kidneys or livers of the animals at days 42 and 165 for all cohorts.

Toxicity of dabrafenib with cisplatin treatment was tested in this study in the kidneys and livers of the treated animals. Combining 2 drugs could pose some systemic toxicity issues; therefore, we wanted to ensure that the combination of dabrafenib and cisplatin was not toxic to major organs that can be damaged from cisplatin. These organs were chosen as it is known that, in addition to the ear, cisplatin accumulates and can cause damage in these tissues (5). No significant damage was recorded by H&E, PAS, and Masson’s trichrome staining in the kidneys or livers of the mice at days 42 and 165 with the cotreatments. Dabrafenib alone, being an FDA-approved drug, was not expected to cause significant damage to the kidneys and livers of the mice in the doses tested in this study, but the toxicity and ototoxicity of the cotreatments were unknown. This demonstration of no significant toxicity or ototoxicity of the drug cotreatments is vital for future clinical trials.

Cisplatin has been shown to accumulate in the inner ear by the Breglio et al. study and may cause long-term hearing loss and possible reduced protection when drug administration does not continue after the cessation of cisplatin treatment (5, 44, 46). For that reason, it is important to test if dabrafenib will protect not only at day 42 when the cisplatin cycles are completed, but also at longer time points, such as 4 months after the treatments. Our results show that dabrafenib-cotreated mice still have significantly better hearing ability compared with cisplatin alone mice. The hearing protection is sustained for up to 4 months following the end of cisplatin treatment, which indicates the protection dabrafenib offers from cisplatin ototoxicity is stable and not acute. Mice only need to be treated with dabrafenib while cisplatin is administered, and more treatments following the cessation of cisplatin are not required to confer protection. This limits the amount of drug patients would need to receive to get optimal hearing protection from dabrafenib.

In the present study, there was no decrease in EP following cisplatin administration, which is contrary to what other studies have found (5, 49–51). We tested EPs at 2 time points following the cisplatin treatment protocol: once after the completion of cycle 3 and once 4 months after cycle 3. In Breglio et al. 2017, the same mouse model and treatment protocol were used, and they observed a 25–30 mV reduction in EP magnitude at the end of cycle 1 and 60 days following cycle 3 (5). These 2 time points were not measured in the current study. However, they also show that there was no decrease in EP when measured at the end of cycle 3; this is difficult to interpret as greater damage to the stria would be expected as cisplatin treatment continued in cycle 3. Breglio et al. 2017 state that the hearing loss and OHC dysfunction can be partially explained by the drop in EP that they observed (5). Based on the present study and others (64, 65), the drop in EP does not seem to be a major causative factor of cisplatin-induced hearing loss and consequent hair cell loss and dysfunction. Hair cell death can occur with a drop in EP, but the decrease in EP observed by Breglio and colleagues is probably not enough to cause hair cell death. Hair cell survival is still observed even when EP is decreased to 18 mV (64), and the study in question shows a decrease to approximately 60–65 mV (5), Additionally, a recent study shows that DPOAEs are normal even when the EP is reduced to 40 mV from 80–100 mV in healthy animals (65). We did not observe any decrease in EP at 4 months following the completion of cycle 3, which demonstrates that cisplatin does not permanently decrease EP, even though it is retained indefinitely in the stria vascularis (5). This, along with the other studies mentioned (64, 65), suggests that any decrease in EP that has been observed following cisplatin administration is not a main causative factor that drives hearing loss and OHC death. Furthermore, these data also suggest that dabrafenib’s protective effect is likely not occurring through protection of the stria vascularis, because strial function appears to be normal despite the fact that cisplatin is retained in stria.

Dabrafenib’s mechanism of protection is not fully understood; however, there are several different cellular pathways that dabrafenib could be exhibiting its protective effect through. Activation of the MAPK pathway is typically associated with cell survival, proliferation, and differentiation, but it has a different role in postmitotic cells, like the inner ear cells. A multitude of studies have demonstrated that activation of this critical pathway induces cell death (25–27, 32). We observe activation of the MAPK pathway in the organ of Corti, and dabrafenib could be preventing hair cell death through inhibition of this pathway. Additionally, activation of the cellular stress MAPK pathway can lead to an increase in reactive oxygen species (ROS) production. Many studies have implicated ROS as a major contributing factor leading to hair cell death and hearing loss following cisplatin treatment (66–69). Inhibition of the MAPK pathway could be preventing this increase in ROS production, which would prevent hair cell death and lower cellular stress. Furthermore, one final potential mechanism that dabrafenib could be exhibiting its protective effect through is the inflammatory and immune cell response. It is well understood that cisplatin causes an increase in cytokines and chemokines, which leads to an increase in immune cells in the cochlea (29, 70–73). These immune cells have been implicated as a possible contributing factor to the hearing loss that occurs following cisplatin treatment (74–77). The MAPK pathway has been shown to alter the immune response and could be exerting its protection from hearing loss through prevention of immune cells’ infiltration (78, 79). Further studies will explore these potential pathways to understand how dabrafenib protects from cisplatin-induced hearing loss.

To conclude, we present in this work promising preclinical results for dabrafenib as a therapeutic candidate for cisplatin-induced hearing loss. It has a low effective dose of one-tenth of the human equivalent dose (3 mg/kg administered twice day), a good toxicity profile, and a therapeutic index of at least 25 in the multidose cisplatin regimen. It protects both female and male mice, reduces hearing loss in 2 strains of mice (FVB/NJ and CBA/CaJ), and offers protection from weight loss that occurs during cisplatin chemotherapy with persistence of hearing protection for at least 4 months after cisplatin treatments. While dabrafenib, an anticancer drug itself, does not interfere with cisplatin’s tumor-killing activity in various lung cancer and neuroblastoma cell lines (6), further animal tumor model studies are needed to establish the best cancer patient population for future clinical trials for hearing protection (13).

## Methods

### Mouse model.

For the single-dose cisplatin protocol, FVB/NJ breeding mice were purchased from The Jackson Laboratory, bred in the animal facility at Creighton University, and used at 6–8 weeks old for the single-dose cisplatin experiment. For the multicycle cisplatin protocol, 8-week-old CBA/CaJ mice were purchased from The Jackson Laboratory with an equal number of males and females. The CBA/CaJ mice were given 1 week to acclimate to the Animal Resource Facilities (ARF) at Creighton University. Animals were anesthetized by Avertin (2,2,2-tribromoethanol) via intraperitoneal injection at a dose of 500 mg/kg, and complete anesthesia was determined via toe pinch. For all experiments, mice were randomly assigned to experimental groups, maintaining a balance of males and females in each group.

### Single-dose cisplatin treatment in mice.

We dissolved 10 mg of cisplatin (479306, MilliporeSigma) powder in 10 mL of sterile saline (0.9% NaCl) at 37°C for 40 to 60 minutes. We administered 30 mg/kg once to FVB mice via intraperitoneal injection on day 1 of the protocol (Figure 1A) (6, 20). One day before cisplatin injection, mice received 1 mL of saline by subcutaneous injection and were given 1 mL of saline twice a day throughout the protocol until body weight started to recover. The cages of cisplatin-treated mice were placed on heating pads until body weights began to recover. Food pellets dipped in DietGel Boost were placed on the cage floor of cisplatin-treated mice. DietGel Boost (72-04-5022 Clear H_2_O) is a high-calorie dietary supplement that provides extra calorie support for mice. The investigators and veterinary staff carefully monitored for changes in overall health and activity that may have resulted from cisplatin treatment.

### Multicycle cisplatin treatment in mice.

We dissolved 4.5 mg of cisplatin (479306, MilliporeSigma) powder in 25 mL of sterile saline (0.9% NaCl) at 37°C for 40 to 60 minutes. We administered 3 mg/kg cisplatin to mice via intraperitoneal injection once a day in the morning. This repeated for 4 total days with a 10-day recovery period in which no cisplatin was administered to the mice. Mice were treated with 3 mg/kg cisplatin for a total of 12 days (4 days per cycle with 3 cycles) (Figure 2A) (46, 47). Cisplatin-treated mice were injected by subcutaneous injection twice a day with 1 mL of warm saline to ameliorate dehydration. This continued until body weight started to recover. The cages of cisplatin-treated mice were placed on heating pads throughout the duration of the experiment until mice began to recover after the third treatment cycle of the protocol. Food pellets dipped in DietGel Boost were placed on the cage floor of cisplatin-treated mice. The investigators and veterinary staff carefully monitored for changes in overall health and activity that may have resulted from cisplatin treatment.

### Compound administration by oral gavage.

The compound dabrafenib mesylate was purchased from MedChemExpress and administered to FVB/NJ and CBA/CaJ mice via oral gavage. Dabrafenib was dissolved in a mixture of 10% DMSO, 5% Tween 80, 40% PEG-E-300, and 45% saline. For the single-dose cisplatin experiment, 12 mg/kg dabrafenib was given to mice once in the morning and once at night. This continued for a total of 3 days (Figure 1A). For the multicycle cisplatin protocol, 15, 3, or 0.6 mg/kg dabrafenib was administered once in the morning and once at night for 4 total days with a 10-day recovery period in which no dabrafenib was administered to the mice. This cycle was repeated for a total of 3 times (Figure 2A). Mice treated with cisplatin and dabrafenib were given dabrafenib 1 hour before treatment with cisplatin in the morning.

### ABR threshold and wave 1 amplitude measurements.

ABR waveforms in anesthetized mice were recorded in a sound booth by using subdermal needles positioned in the skull, below the pinna, and at the base of the tail, and the responses were fed into a low-impedance Medusa digital biological amplifier system (RA4L; TDT; 20 dB gain). At the tested frequencies (8, 16, and 32 kHz), the stimulus intensity was reduced in 10 dB steps from 90 to 10 dB to determine the hearing threshold. ABR waveforms were averaged in response to 500 tone bursts with the recorded signals filtered by a band-pass filter from 300 Hz to 3 kHz. ABR threshold was determined by the presence of at least 3 of the 5 waveform peaks (6, 20). Baseline ABR recordings before any treatment were performed when mice were 6–7 weeks old for the single-dose cisplatin experiments and 9 weeks old for the multidose cisplatin protocol. All beginning threshold values were between 10 and 40 dB at all tested frequencies. In the single-dose cisplatin experiment, posttreatment recordings were performed 21 days following cisplatin treatment. For the multicycle cisplatin protocol, posttreatment recordings were performed 42 days after the start of the 3-cycle protocol (aged 18 weeks) with half the mice kept alive, and ABR was performed again on these mice 4 months after the completion of the 42-day treatment protocol. All thresholds were determined independently by 2–3 experimenters for each mouse, who did not know the treatment the mice received. ABR wave 1 amplitudes were measured as the difference between the peak of wave 1 and the noise floor of the ABR trace.

### DPOAE measurements.

DPOAEs were recorded in a sound booth while mice were anesthetized. DPOAE measurements were recorded using the TDT RZ6 processor and BioSigTZ software. The ER10B+ microphone system was inserted into the ear canal in a way that allowed for the path to the tympanic membrane to be unobstructed. DPOAE measurements occurred at 8, 16, and 32 kHz with an f2/f1 ratio of 1.2. Tone 1 was ×0.909 of the center frequency, and tone 2 was ×1.09 of the center frequency. DPOAE data were recorded every 20.97 ms and average 512 times at each intensity level and frequency. At each tested frequency, the stimulus intensity was reduced in 10 dB steps starting at 90 dB and ending at 10 dB. DPOAE threshold was determined by the presence of an emission above the noise floor. Baseline DPOAE recordings occurred when CBA/CaJ mice were 10 weeks old with testing repeated on day 42 (immediately after cycle 3) and on day 165 (4 months after cycle 3). DPOAE threshold shifts were determined by subtracting the baseline DPOAE recording from the postexperimental recording.

### Tissue preparation, immunofluorescence, and OHC counts.

Cochleae from adult mice were prepared and examined as described previously (80–82). Cochleae samples were immunostained with anti–myosin VI (1:400; 25-6791, Proteus Bioscience) or anti–phosphorylated ERK antibody (1:400; 9101L, Cell Signaling Technology) with secondary antibodies purchased from Invitrogen coupled to anti-rabbit Alexa Fluor 488 (1:400; A11034). All images were acquired with a confocal microscope (LSM 700 or 710, Zeiss). OHC counts were determined by the total number of OHCs in a 160 μm region (6, 20, 82). Counts were determined for the 8, 16, and 32 kHz regions. Cochleae from each experimental group were randomly selected to be imaged for OHC counts.

### EP measurements.

Mice were anesthetized using a combined regimen of ketamine (16.6 mg/mL) and xylazine (2.3 mg/mL) and supplemented as needed to maintain a surgical level via intraperitoneal injection. For recording the EP, a round-window approach was used. A glass capillary pipette electrode (10 MU) was mounted on a hydraulic micromanipulator and advanced until a stable positive potential was observed. Signals were filtered and amplified under current-clamp mode using an Axopatch 200B amplifier (Molecular Devices) and acquired by software pClamp 9.2. The sampling frequency was 10 kHz (52, 53, 64).

### Kidney histology examination.

Following cisplatin and dabrafenib treatment, mice were sacrificed, and kidneys were extracted and put into 4% paraformaldehyde (PFA). The kidneys were later embedded in paraffin, sectioned (3 μm), and stained with H&E and PAS. Sections were observed under a microscope (Nikon Eclipse Ci) for histological examination. A semiquantitative pathological scoring system was used as described in Pabla et al., 2015, and Hu et al., 2010 (54, 55). The grading system uses scores 0–4 that indicate the percentage of damage in each section. Sections were analyzed by an experienced pathologist in a double-blind manner. The grades are: grade 0 (minimal) = < 10% damage with no visible lesions and normal morphology; grade 1 (mild) = 11%–25% damage with mild tubule dilation, swelling of cells, presence of luminal debris or cast, and nuclear condensation with partial loss of brush borders in one-third of tubules; grade 2 (moderate) = 26%–50% damage with clear dilation of tubules, loss of brush borders, nuclear loss, and presence of casts in less than two-thirds of tubules; grade 3 (marked) = 51%–75% damage with severe dilation of most tubules, total loss of brush borders, and nuclear loss in two-thirds of tubules; and grade 4 (severe) = > 75% damage with complete loss of tissue morphology, severe tubule dilation, and loss of nucleus and brush borders.

### Liver histology examination.

Following cisplatin and dabrafenib treatment, mice were sacrificed, and livers were extracted and put into 4% PFA. The livers were later embedded in paraffin, sectioned (3 μm), and stained with H&E and Masson’s trichrome stain. Sections were observed under a microscope (Nikon Eclipse Ci) for histological examination. The grading system uses a score of 0–4 that indicates the amount of damage in each section. Sections were analyzed by an experienced pathologist in a double-blind manner. The grades are grade 0 (normal), grade 1 (mild damage), grade 2 (moderate damage), grade 3 (severe damage), and grade 4 (very severe/fulminant damage). Criteria that determined the scoring of each liver sample was the presence of fibrosis, lobular disarray, hepatocyte swelling, hepatocyte nuclear changes, hepatocyte necrosis, lobular inflammation, portal inflammation, sinusoidal and central vein congestion, and Kupffer cell hyperplasia (56, 57).

### Statistics.

Statistical analysis was performed using Prism (GraphPad Software). Two-way ANOVA with Bonferroni’s post hoc test was used to determine mean difference and statistical significance. Statistical significance was determined when *P* < 0.05.

### Study approval.

All animal experiments included in this study were approved by Creighton University’s Institutional Animal Care and Use Committee in accordance with policies established by the Animal Welfare Act and Public Health Service.

### Data availability.

All data needed to evaluate the conclusions in the paper are present in the paper or supplement. The raw data are available in the Excel file provided in the supplement titled “Supporting Data Values.”

## Author contributions

TT conceived the project. MAI, RDL, RGK, MTM, and TT designed and performed in vivo experiments. HL and DZZH performed and analyzed the EP measurements. CKP and MAI performed staining and preparation of histological samples. WJH analyzed and scored kidney and liver tissues. MAI performed cochlear dissection and confocal imaging. TT, MAI, and RDL contributed to experimental design and data analysis. TT, RDL, and MAI wrote the manuscript with input from all coauthors. Co–first authors contributed equally to the study and are listed in alphabetical order by last name.

## Supplementary Material

Supplemental data

Supporting data values

## Version 1 11/07/2023

In-Press Preview

## Version 2 12/22/2023

Electronic publication
