# Supplementary material for: Dabrafenib protects from cisplatin-induced hearing loss in a clinically relevant mouse model
Source: JCI Insight. 2023 Dec 22;8(24):e171140. doi: 10.1172/jci.insight.171140 (PMC10807719; doi:10.1172/jci.insight.171140)
Supplement: Supplemental data [file jciinsight-8-171140-s009.pdf]

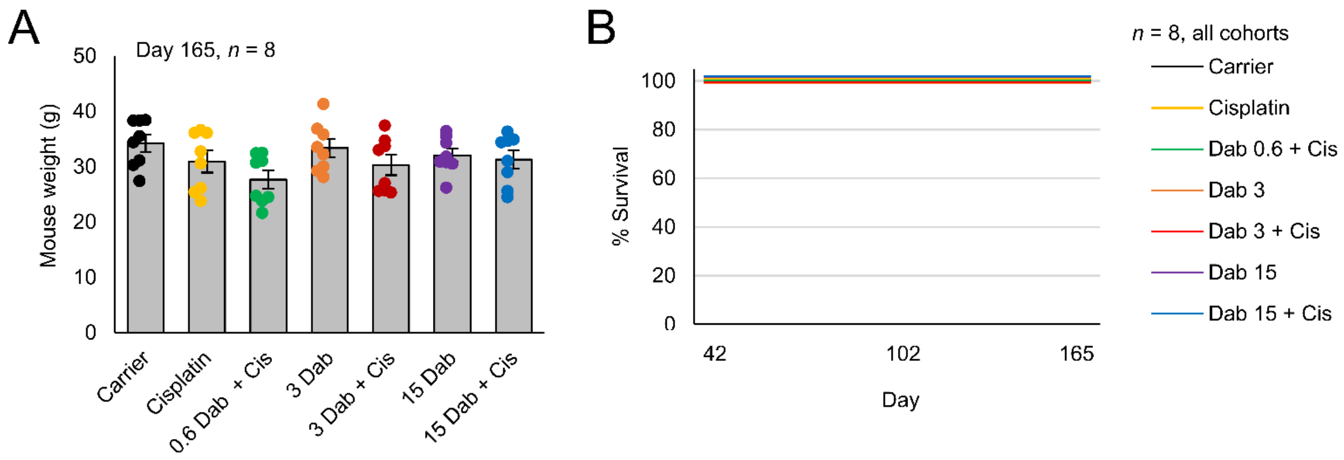


**<u>Supplemental Figure 1:</u> No differences in weight or survival was observed at Day 165. (A)** Mouse weights at day 165 of treatment protocol in Figure 2A. **(B)** Kaplan-Meier survival curves of mouse cohorts going to day 165. Carrier (black), cisplatin alone (yellow), 15 mg/kg dabrafenib alone (purple), 3 mg/kg dabrafenib alone (orange), 15 mg/kg dabrafenib plus cisplatin (blue), 3 mg/kg dabrafenib plus cisplatin (red), and 0.6 mg/kg dabrafenib plus cisplatin (green).

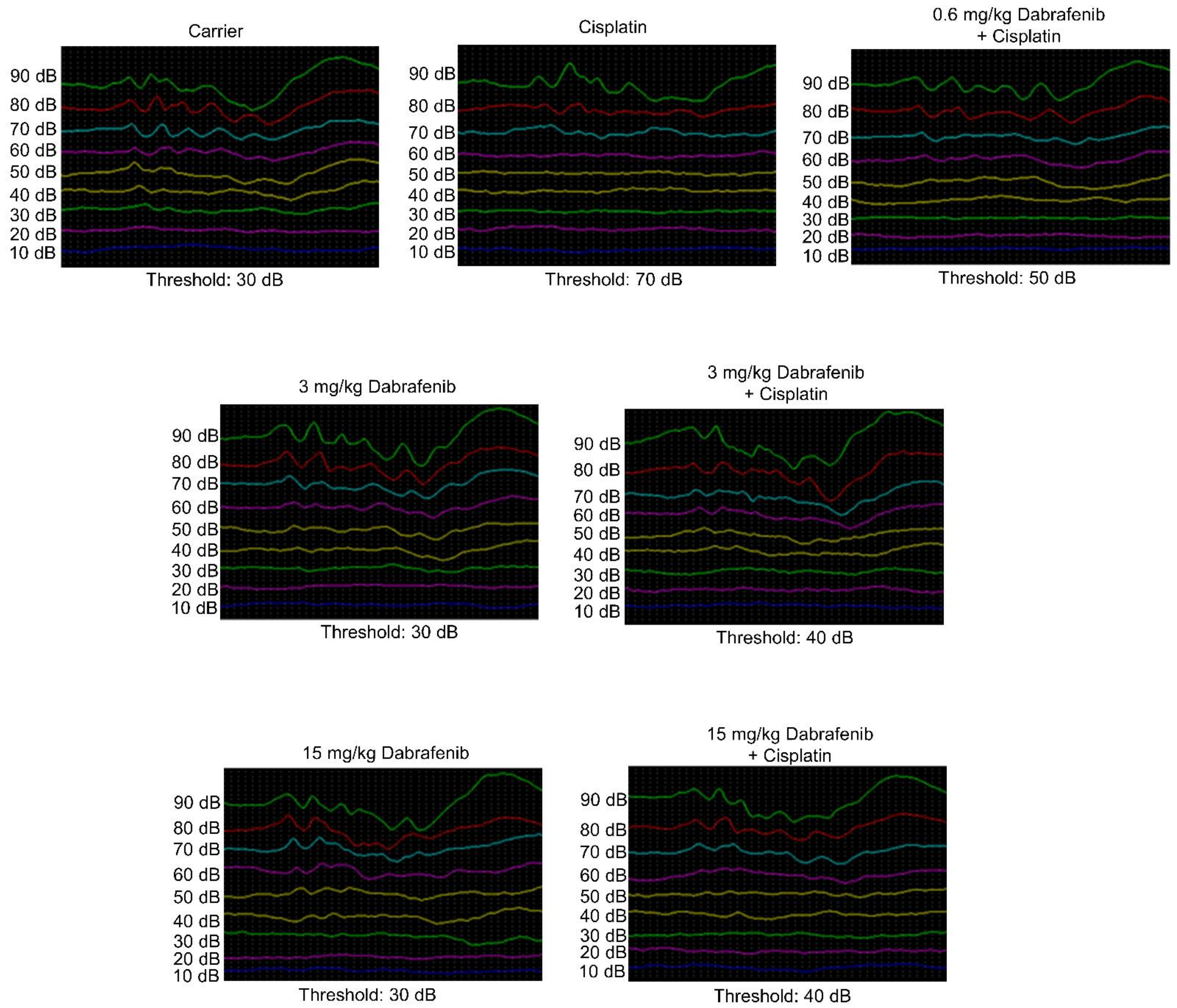


**<u>Supplemental Figure 2:</u> Representative ABR trace recordings, related to figure 3.** Representative post treatment ABR recordings from mice treated with carrier alone, cisplatin alone, 15 mg/kg dabrafenib alone, 3 mg/kg dabrafenib alone, 15 mg/kg dabrafenib and cisplatin co-treatment, 3 mg/kg dabrafenib and cisplatin co-treatment, and 0.6 mg/kg dabrafenib and cisplatin co-treatment. The ABR traces are from the experiment in Figure 3 and the threshold was recorded as the last trace with at least 3 of the 5 ABR waveforms present.

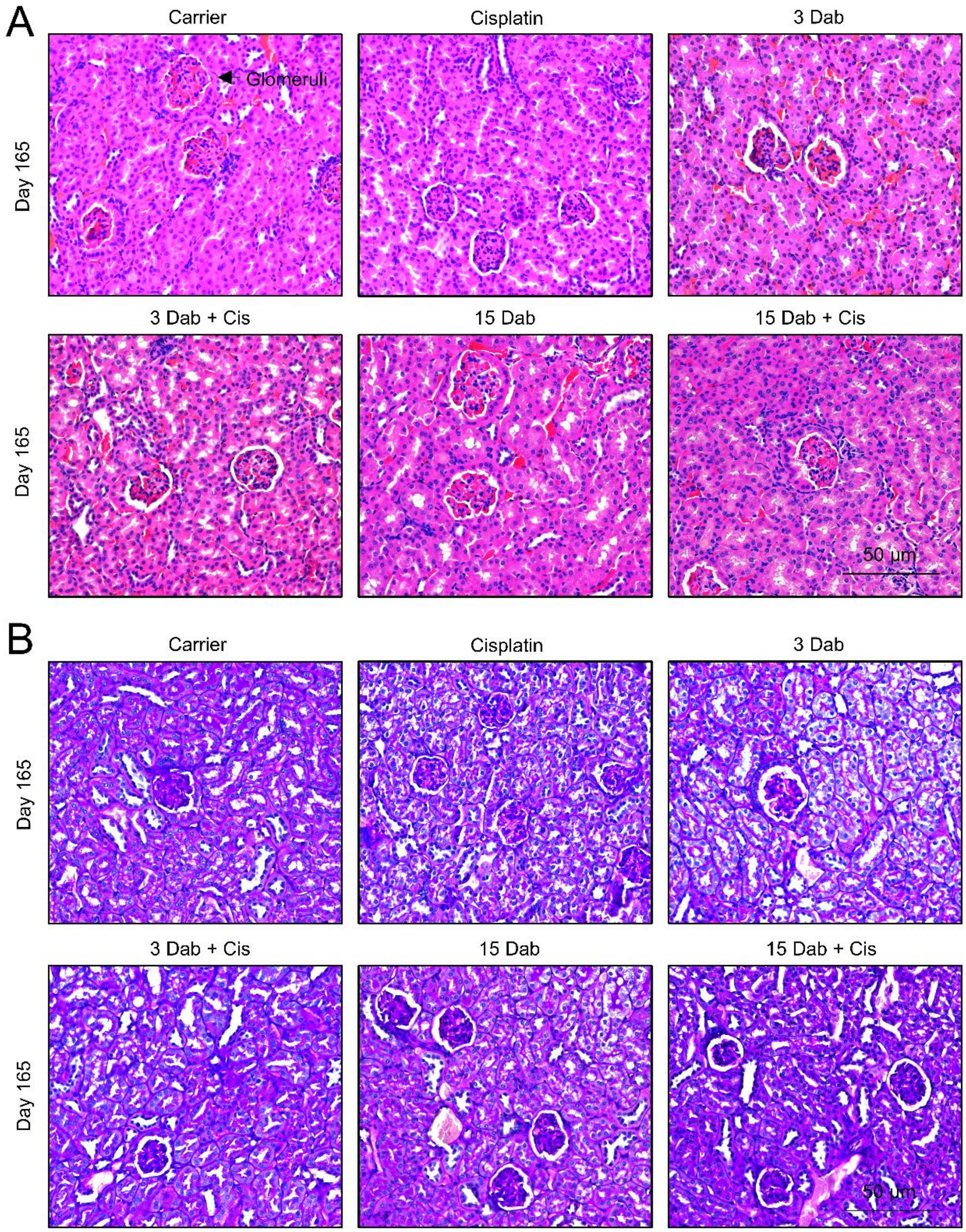


**<u>Supplemental Figure 3:</u> Representative images of kidneys dissected and stained at day 165 of the protocol. (A)** Representative H&E stained images of the kidney when dissected and stained at day 165 (4-months post cisplatin treatment). The 6 treatment groups that were analyzed are carrier alone, cisplatin alone, 15 mg/kg dabrafenib alone, 3 mg/kg dabrafenib alone, 15 mg/kg dabrafenib and cisplatin co-treatment, and 3 mg/kg dabrafenib and cisplatin co-treatment. **(B)** Representative PAS stained images of the kidney when dissected and stained at day 165.

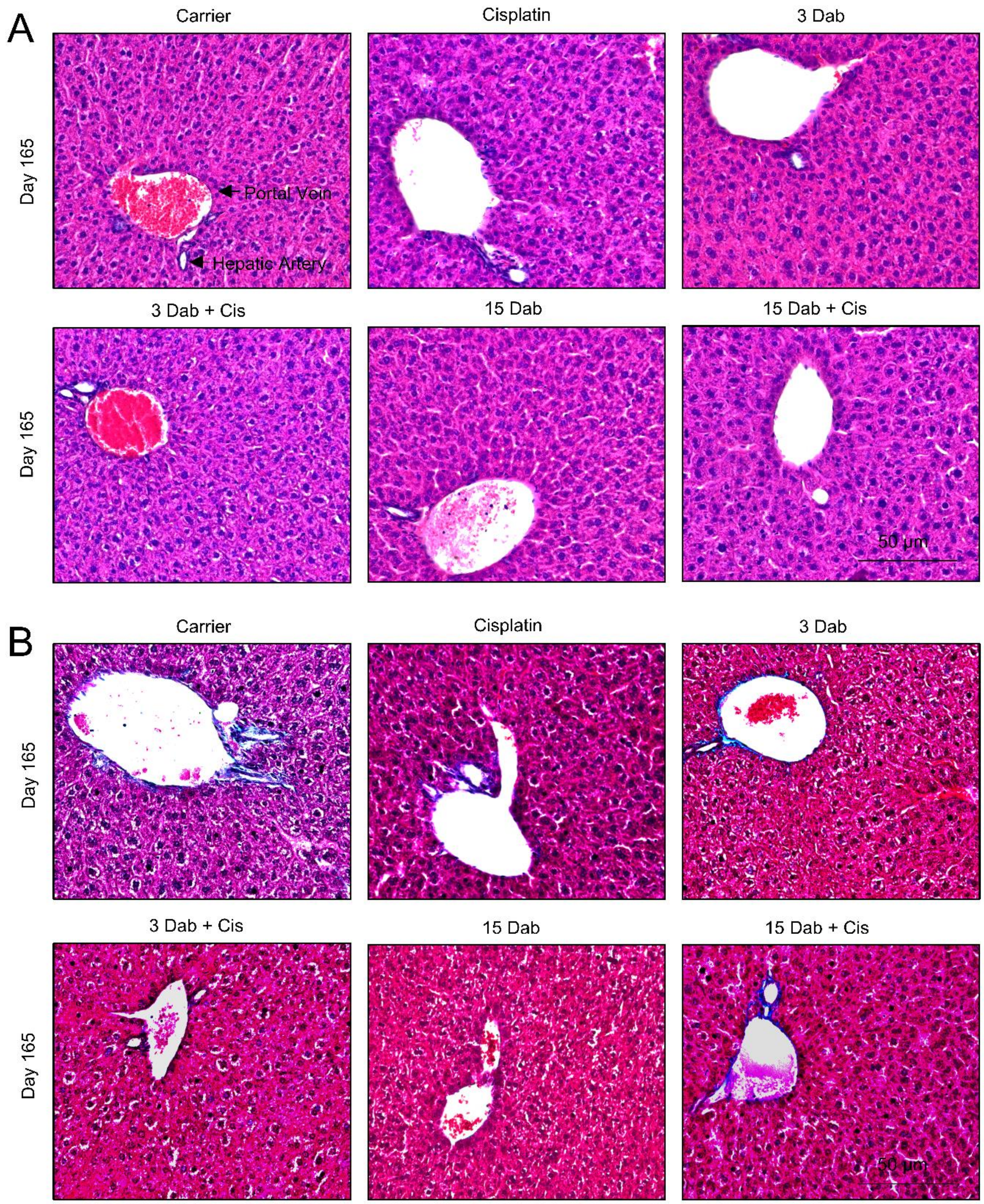


**<u>Supplemental Figure 4:</u> Representative images of livers dissected and stained at day 165 of the protocol. (A)** Representative H&E stained images of the liver when dissected and stained at day 165 (4-months post cisplatin treatment). The 6 treatment groups analyzed are the following: carrier alone, cisplatin alone, 15 mg/kg dabrafenib alone, 3 mg/kg dabrafenib alone, 15 mg/kg dabrafenib and cisplatin co-treatment, and 3 mg/kg dabrafenib and cisplatin co-treatment. **(B)** Representative Masson's Trichrome stained images of the liver at day 165 of experimental protocol.
